# Meaningfully meeting the interoperability mandate: a review of the Assistant Secretary for Technology Policy Real World Testing practices

**DOI:** 10.1093/jamiaopen/ooaf044

**Published:** 2025-06-11

**Authors:** Jessica L Handley, Alicia Farlese, Sophia Lager, Ajit A Dhavle, Shahzad Ahmad, Anna Mathias, Raj M Ratwani

**Affiliations:** MedStar Health National Center for Human Factors in Healthcare, Washington, DC, 20008, United States; HealtheConsulting, Lexington, KY, 40502, United States; Colgate University, Hamilton, NY, 13346, United States; Quandary Peak, Los Angeles, CA, 90012, United States; Quandary Peak, Los Angeles, CA, 90012, United States; Quandary Peak, Los Angeles, CA, 90012, United States; MedStar Health National Center for Human Factors in Healthcare, Washington, DC, 20008, United States; Georgetown University School of Medicine, Washington, DC, 20057, United States

**Keywords:** electronic health records, interoperability, policy

## Abstract

**Objectives:**

We analyzed interoperability-related Real World Testing results to identify whether developers are providing meaningful results with the appropriate context to enable stakeholders to understand the Certified Health IT conformance and interoperability when deployed in production environments.

**Materials and Methods:**

This qualitative study analyzed components of the Assistant Secretary for Technology Policy’s transitions of care criterion Real World Testing results of 5 inpatient and 5 ambulatory health IT developers with the largest market share.

**Results:**

Developers provided interoperability measures; however, none of the developers’ presented results in a meaningful way with the appropriate context to understand product interoperability.

**Discussion:**

Our results suggest that developers with ASTP/Office of the National Coordinator (ONC) Certified Health IT modules are not providing interoperability transparency through Real World Testing as required by the ONC Health IT Certification Program and intended by the 21st Century Cures Act.

**Conclusion:**

Clearer developer guidance and actual metric requirements on Real World Testing may be required and the authorized certification bodies, who review developer results, may need to more closely inspect reports to look at the quality of reported results.

## Background and significance

Health information technology (IT) interoperability is critical for safe, effective, and efficient care.^1^ Interoperability enables authorized access to, and usage of, all electronically accessible health information, and secure exchange of electronic health information with other health IT without special effort on the part of the user. The Assistant Secretary for Technology Policy/Office of the National Coordinator for Health Information Technology (hereafter ASTP) has requirements in place to promote interoperability through its Health IT Certification Program. Despite these requirements, interoperability has been a longstanding challenge for health-care facilities, providers, patients, and other health-care stakeholders.^2^

The 21st Century Cures Act sought to address these ongoing challenges by requiring the ASTP’s Health IT Certification Program to expand its scope to now include Conditions and Maintenance of Certification requirements to ensure ONC Certified Health IT products perform as intended with respect to functionality and interoperability requirements in actual clinical settings. Developers of ONC Certified Health IT are now required to demonstrate, in a public and transparent way, that their products operate in the intended “real world” settings while adhering to the certification’s interoperability and functionality requirements. To meet this requirement, developers submit a Real World Testing Plan annually to their ONC-Authorized Certification Body (ONC-ACB), conduct the proposed testing, and report the results. Office of the National Coordinator-Authorized Certification Bodies are to conduct a “completeness review” of these annual real world test plans and ensure they, and the corresponding testing results, are made publicly available.^3^

The ASTP has several different Real World Testing criteria for interoperability. Developers of ONC Certified Health IT are required to plan for and test all real world criteria that are applicable to their product’s scope of certification for all settings in which their product is marketed and may use a single measure to meet the criteria.^4^ Neither the ASTP nor the ONC-ACB specifies the developer testing methodologies or outcome measures. Rather, flexibility is provided for developers to specify methods and outcome measures that are best for their products and clinical settings. Historically, in response to certification requirements, many developers have provided incomplete/inaccurate information and/or testing results that do not enable meaningful assessment of the product’s functions and capabilities for which they are certified.^5^^,^^6^

## Objectives

We analyzed 2022 and 2023 Real World Testing results from developers of ONC Certified Health IT with the largest market share to assess whether their reported measure(s) provide meaningful information that would enable a health-care provider, facility, or other consumer of the report to assess the products interoperability and conformance in a real world setting.

## Materials and methods

Our analysis focused on the 2015 Edition 21st Century Cures updated “transitions of care” certification criterion given its essential role in the interoperable exchange of electronic health information. The 2022 and 2023 Real World Testing results reports, made available in 2023 and 2024, respectively, were retrieved from the Certified Health Product List for the inpatient (ie, Epic, Oracle Cerner, MEDITECH, CPSI, Allscripts) and 5 ambulatory (ie, Epic, eClinicalWorks, athenahealth, Allscripts, NextGen) ONC Certified Health IT developers with the largest market share, resulting in a total of 8 unique developers.^7^^,^^8^ It should be noted that for the 2023 Real World Testing results, Allscripts (now Veradigm) is an ambulatory developer only resulting in only 4 inpatient developers for analysis in 2023. Health IT developers with products certified to the ONC health IT certification criterion “transitions of care” must remain conformant with the functionality and interoperability requirements to send, receive, validate, display, and create care transitions/referral summaries. Per ONC Health IT Certification Program Testing Resource Guide, developers are encouraged to provide a denominator when reporting results to provide full transparency and context on how many documents were attempted during send/receive. This guidance is reflective of the criterion’s requirement to detect and review valid and invalid Consolidated Clinical Document Architectures (C-CDAs) as well as Centers for Medicare & Medicaid Services interoperability program requirements for both the numerator and denominator enabled by certified health IT.^9^ Without this information, the reader would not be able to determine interoperability challenges and/or success.^10^ Our analysis focused on the send and receive, and validate and display components of this criterion’s requirement since these are good indicators of compliance with the full criterion. We coded whether, (1) any information was reported related to the interoperability criterion and, (2) whether the information was presented in a meaningful way such that a report reader could determine interoperability success/failure. This would involve using a measure that reflects number of successful/failed exchanges relative to the total number of attempted exchanges. The specific ONC Certification Program requirements for this care coordination relation criterion, transitions of care, are summarized in Table 1.

**Table 1.** Transitions of care criterion by regulation section.

Transitions of care regulation sections	Certification requirement components
Send and receive via edge protocol	Send transition of care/referral summaries through a method that conforms to the standard and leads to their processing
	Receive transition of care/referral summaries through a method that conforms to the standard from a standard implementation
	Receive and make available the contents of an Experience Data Model (XDM) package
Validate and display	Demonstrate the ability to detect valid and invalid transition of care/referral summaries received
	Display in human readable format the data included in transition of care/referral summaries received
	Allow for the individual display of each section (and the accompanying document header information) that is included in a transition of care/referral summary received

Three researchers independently reviewed the Real World Testing reports for each of the developers per year and used the § 170.315 (b)(1) *Transitions of care* regulation criterion to determine whether developers reported meaningful measures of interoperability. Following independent review, the researchers’ compared findings via Google Sheets. To organize and interpret the data, we used a framework analysis, and discrepancies were discussed to reach resolution through consensus.^11^

## Results

In 2022, 6 developers (of 8, 75%) and in 2023, 5 developers (62.5%) provided information regarding sending data. In 2022 and 2023, 7 developers (87.5%) provided information regarding receiving data, mostly reporting total counts of sent and received messages (Table 2). In 2022 and 2023, 2 developers (25%) provided information related to validating documents and 1 developer (12.5%) provided information of display criteria. None of the developers provided information regarding Experience Data Model processing or displaying section views in 2022 or 2023. In 2022 and 2023, none of the 8 health IT developers provided Real World Testing results in a way that meaningfully demonstrated real world interoperability and functionality relative to the transitions of care criterion requirements and intent of the Real World Testing paradigm.

**Table 2.** Information provided by Certified Health IT developers for transitions of care criteria.

Year	Certified Health IT developer (product)		Send and receive via edge protocol			Validate and display		
			Sending transition of care/referral summaries	Receiving transition of care/referral summaries	Receiving and making available the contents of a XDM package	Detect valid and invalid transition of care/referral summaries received	Displaying in human readable format the data included in transition of care/referral summaries received	Allowing for the individual display of each section (and the accompanying document header information) that is included in a transition of care/referral summary received
2022	Allscripts (professional EHR)^a^	Information provided	Yes (counts of successfully transmitted C-CDAs and counts of failed C-CDA transmissions)	Yes (counts of received C-CDAs)	No	No	No	No
		Meaningful information provided	No	No	No	No	No	No
	Athenahealth (athenaClinicals)^b^	Information provided	Yes (counts of C-CDAs successfully sent and counts of failures)	Yes (counts of C-CDAs successfully received and counts of failures)	No	No	No	No
		Meaningful information provided	No	No	No	No	No	No
	Computer Programs and Systems, Inc. (CPSI) (Centriq)^c^	Information provided	Yes (counts of C-CDAs sent)	Yes (counts of C-CDAs received)	No	No	No	No
		Meaningful information provided	No	No	No	No	No	No
	eClinicalWorks (eClinicalWorks)^b^	Information provided	Yes (counts of messages sent)	Yes (counts of messages received)	No	No	No	No
		Meaningful information provided	No	No	No	No	No	No
	Epic Systems Corporation (EpicCare Inpatient Base and EpicCare Ambulatory Base)^a^	Information provided	Yes (counts of messages sent, counts of all messages sent, and ratio of all messages sent relative to all visits)	Yes (counts of messages received, counts of all messages received, and ratio of all messages received relative to all visits)	No	No	No	No
		Meaningful information provided	No	No	No	No	No	No
	Medical Information Technology, Inc. (MEDITECH)^c^	Information provided	Yes (success rate of sent messages for C-CDA files)	Yes (count of accepted messages and success rate of accepted messages for C-CDA files)	No	No	No	No
		Meaningful information provided	No	No	No	No	No	No
	NextGen (NextGen Ambulatory EHR)^b^	Information provided	No	Yes (counts of imported C-CDA documents)	No	Yes (percentages of validated successes and failures)	No	No
		Meaningful information provided	No	No	No	No	No	No
	Oracle Cerner (PowerChart)^c^	Information provided	No	No	No	Yes (average number of times the C-CDA validator capability was leveraged per month)	Yes (average number of times a C-CDA document was opened/viewed per month)	No
		Meaningful information provided	No	No	No	No	No	No
2023	Veradigm EHR (formerly Allscripts Professional EHR)^b^	Information provided	Yes (counts of successfully transmitted C-CDAs and counts of failed C-CDA transmissions)	Yes (counts of received C-CDAs)	No	No	No	No
		Meaningful information provided	No	No	No	No	No	No
	Athenahealth (athenaClinicals)^b^	Information provided	Yes (counts of C-CDAs successfully sent and counts of failures)	Yes (counts of C-CDAs successfully received and counts of failures)	No	No	No	No
		Meaningful information provided	No	No	No	No	No	No
	TruBridge (Centriq) (Formerly CPSI Centriq)^c^	Information provided	Yes (counts of C-CDAs sent)	Yes (counts of C-CDAs received)	No	No	No	No
		Meaningful information provided	No	No	No	No	No	No
	eClinicalWorks (eClinicalWorks)^b^	Information provided	Yes (counts of messages sent)	Yes (counts of messages received)	No	No	No	No
		Meaningful information provided	No	No	No	No	No	No
	Epic Systems Corporation (EpicCare Inpatient Base and EpicCare Ambulatory Base)^a^	Information provided	Yes (counts of messages sent, counts of all messages sent, and ratio of all messages sent relative to all visits)	Yes (counts of messages received, counts of all messages received, and ratio of all messages received relative to all visits)	No	No	No	No
		Meaningful information provided	No	No	No	No	No	No
	Medical Information Technology, Inc. (MEDITECH)^c^	Information provided	Yes (success rate of sent messages for C-CDA files)	Yes (count of accepted messages and success rate of accepted messages for C-CDA files)	No	No	No	No
		Meaningful information provided	No	No	No	No	No	No
	NextGen (NextGen Ambulatory EHR)^b^	Information provided	No	Yes (counts of imported C-CDA documents)	No	Yes (percentages of validated successes and failures)	No	No
		Meaningful information provided	No	No	No	No	No	No
	Oracle Cerner (Millennium)^c^	Information provided	No	No	No	Yes (average number of times the C-CDA validator capability was leveraged per month)	Yes (average number of times a C-CDA document was opened/viewed per month)	No
		Meaningful information provided	No	No	No	No	No	No

Abbreviations: EHR, electronic health record; XDM, Experience Data Model.

a Signifies both inpatient and ambulatory developers.

b Signifies ambulatory developers.

c Signifies inpatient developers.

## Discussion

Developers of ONC Certified Health IT with the largest market share generally did not report transitions of care Real World Testing results in a meaningful way. There were no significant differences between the results of inpatient and ambulatory care testing of the electronic health record (EHR) developers in 2022 or 2023. When developers are providing measures, they do not enable understanding of whether the product demonstrates interoperability in “real world” settings. Congress’ intent in the 21st Century Cures Act was to provide greater transparency on product interoperability to inform consumers, patients, providers, and policy makers. Our analysis suggests this intent is not being met.

To address this, the ASTP and the ONC Health IT Certification Program should promptly instruct ONC-ACBs to inspect Real World Testing requirement results to determine whether the measures being used are meaningful for conveying information about the health IT product’s interoperability and conformance to the full scope of the certification criteria as required. This analysis and a previous analysis demonstrating lack of developer adherence to usability certification requirements suggests that an ONC-ACB completeness review that does not inspect the rigor and quality of developers meeting the certification requirements may be problematic.^12^ Additionally, the ASTP should consider future rulemaking requiring developers to provide specific and normalized data for comparable evidence of Real World Testing demonstrating their health IT continues to meet ONC certification requirements and having ONC-ACBs inspect the provided evidence for continued compliance. There have been several legal settlements with health IT developers for ONC Health IT Certification Program compliance-related violations and improving the rigor of the certification adherence process, as well as postmarket surveillance may help address these real world, “in the field” ONC Certified Health IT compliance issues.^13^

Additionally, the ASTP should work to develop prescriptive and detailed guidance complete with best practice recommendations and examples to not only assist developers in their creation and execution of testing plans but can also serve as an important reference document to aid the ONC-ACBs. The ONC-ACBs should provide specific templates for developers to complete to provide greater structure and comparability of Real World Testing results. The 21st Century Cures Act also called for the creation of an EHR Reporting Program to provide transparent reporting on several categories, including interoperability. This is being implemented as the “Insights Condition” and Maintenance of Certification within the ONC Health IT Certification Program. Developers will have specific metrics defined in regulation that developers must report on beginning in 2027. In several years, the “clinical care information exchange” measure may address some of the issues identified in our analysis.

The expectation from Real World Testing was that developers would find conformance issues when conducting their testing, report these issues to their ONC-ACB for transparency, and work to remedy these issues. Policy makers may want to consider establishing an independent organization to conduct proactive surveillance of ONC Certified Health IT products to ensure real world conformance with certification requirements. This would involve independent bodies reviewing real world functioning of EHR’s with certified capabilities rather relying on developer testing reports. Historically, the ONC Health IT Certification Programs has had ONC-ACBs conduct surveillance; however, this effort was underresourced and the ONC-ACBs rely on developers as their customers which may introduce bias in the surveillance process. Budgeting for these types of efforts continues to be a challenge.

There are limitations to this study. Our analysis was focused on the transitions of care criteria. There are other Real World Testing criteria that we did not examine, and it is possible that developers provided meaningful measures aligned with those criteria. Our analysis was limited to the 5 inpatient and 5 ambulatory Certified Health IT products with the largest market share which may not represent all developers of ONC Certified Health IT and other developers may be adhering to the ONC certification requirements. Finally, the analysis was limited to what we could extract from the publicly available Real World Testing reports.

## Conclusion

Our results suggest developers of ONC Certified Health IT are not providing transparent evidence through Real World Testing that their products conform to interoperability certification requirements as intended by the 21st Century Cures Act. We suggest that additional Real World Testing guidance and regulatory requirements appear necessary for developers to demonstrate, and their ONC-ACBs to ensure, in a meaningful and transparent way, that the ONC Certified Health IT developers meet interoperability and functionality requirements in real world settings.

## Data Availability

The data underlying this article include 2022 and 2023 Real World Testing result reports and are publicly available from the Assistant Secretary for Technology Policy at https://www.healthit.gov/topic/certification-ehrs/real-world-testing.
